# Human Temperaments. V.—The Faddist


**Published:** 1916-06-24

**Authors:** 


### HUMAN TEMPERAMENTS.

### V.—The Faddist.*

The faddist, or crank, is familiar to everyone. He is familiar because he insists on making himself heard and seen. He is a busy, meddling, intrusive person, who is not content merely to live and let live, but insists that everyone else should live as he would have them live; in short, should adopt his fad. The faddist is a person who fixes upon some minor phase of conduct and exalts the cult of this mode of conduct into a religion, nay, into more than a religion; for with most people religion nowadays occupies a very subordinate position among the objects for which life is lived, but the faddist lives mainly for his fad. He devotes the greater part of his life to its practice and advocacy, and to abuse and detraction of those who refuse to worship the god of his idolatry.

### Positive and Negative Fads.

There are many fads and many kinds of fads, some of them positive—that is to say, inculcating a certain practice; others negative—that is to say, opposing a certain practice. Many of them are of very trivial importance, and none of them has a tithe of the importance that is attributed to it by the faddist. Of the positive fads the most numerous are those which are supposed, on very insufficient evidence, to contribute to health. The health faddist eats strange food. He lives on fruit and nuts or toast and water; his fruit and nuts, or whatever the faddy food is, must come from a particular source of supply and be of a particular brand. His beverage must be measured to the thimbleful. If he drinks tea, it must draw for so many seconds by a stop-watch, and the milk must be sterilised. He wears strange clothes; he must or must not wear certain fabrics next his skin; his clothes must be of special material, of a special brand, and must be made by a certain tailor and no other. He wears no hat; or his hat must be of faddy shape or faddy material, or both. He has a certain ritual of exercises: breathing exercises, exercises to strengthen the lungs, the heart, the stomach, the liver, the arms, the legs, and the rest of him; and this ritual he performs daily with all the zeal of a religious devotee, for to him it is a religion. He must chew each mouthful so many times, and cannot talk at meals because he is engaged in counting the movements of his jaws. All this he does to preserve a life that is not worth preserving; to keep in health a body that serves no useful purpose. His motto is *Propter vitam perdere vitæ causas.*

The negative fads are still more numerous and diversified. The negative faddist is anti-alcoholic, anti-carnivorous, anti-tobacconist, anti-vaccinationist, anti-vivisectionist, anti-patriotist, anti-bellumist; he is anti-restraintist, and would abolish the imprisonment of criminals and the confinement of lunatics. If criminals must be imprisoned, in spite of his efforts to prevent it, they must at any rate be pampered. They must be allowed to have pianos and newspapers, beer and skittles. He is anti-scientist or anti-Christian; he is an astrologer, a neoteric Buddhist, a flat-earth crank, a spiritualist, a telepathist; in short, the faddist holds any opinion and advocates any cause of action you please so long as it is opposed to the general sense of the community.

### The Nature of Fads.

Some of the opinions of the faddist might be held on reasoned conviction, and after examination of the evidence for and against; but it is not thus that the faddist holds them; and when so held they are not fads. The convictions of the faddist, if convictions they can be called, are the merest prejudices. Anything that runs contrary to generally received opinion appeals to him with irresistible force, and he is ready to adopt it without any consideration of the evidence: in fact, he is constitutionally incapable of weighing evidence, of suspending his judgment, or of entertaining doubts. If he has any knowledge at all that there are reasons against his opinion, he gains this knowledge from those who are already steeped in prejudice against the reasons, and take care to put them so as to render them ridiculous. The faddist is incapable of stating fairly an opinion contrary to his own or a fact that traverses his opinion. He garbles, misquotes, distorts, and misrepresents facts, quotations, and opinions that are distasteful to him; and when these expedients will not serve, he suppresses the evidence altogether. What reasons he gives for his faith are childish, but he is incapable of recognising their inconclusive character. The anti-alcoholic, for instance, is never tired of proclaiming that alcohol is not a food, and this is one of his stock arguments against the consumption of alcohol. When he has said that alcohol is not a food he sits down complacently with the conviction that he has said the last and the decisive word. Draw his attention to the fact that the same may be said of tea and coffee, of ginger-beer and lemonade, and even of water, and you incur his detestation and abuse, but you do not shake his confidence in the conclusiveness of his argument.

### The Conscience of the Faddist.

The faddist has a very remarkable conscience. To any infraction of the fad his conscience is morbidly sensitive, it quivers with hyperæsthesia; but in the treatment of the adversaries of his fad it is shockingly callous. Rather than permit the slightest infraction of his fad he is willing to sacrifice the lives of his wife and children, and even, within limits, his own comfort. One distinguished anti-alcoholic faddist electrified the world by proclaiming that if his wife were dying and a drop of alcohol

* The previous articles appeared on May 6, 13, 27, and June 10, 1916; and a letter on the subject on May 20.

would save her life, he would not allow her to have it. He did not make the same promise for himself, if he should be in the same desperate condition; but then, of course, an expedient may be permitted to save the life of a dignitary of the Church that would be very improper if utilised for a mere woman, and that woman only his wife. Such an extreme sacrifice must not be expected; but it is certain that a faddist would put up with inconvenience, even considerable inconvenience, for himself, rather than taste alcohol, or eat meat, or wear an undergarment of cotton or linen or woollen, or infringe the sanctity of his fad, whatever it happened to be. But the sensitiveness of his conscience is in this direction balanced by its extraordinary callousness in treating opposition to his fad. The fad is to him the main end of life, and justifies any means that he may employ to uphold it, and to damage those who are opposed to it. In support of his fad the faddist will conscientiously lie, perjure himself, slander and traduce his antagonists with eagerness and relish. Recognising that the best mode of defence is counter-attack, the faddist considers it more important to carry the war into the enemy's country than even to spread his own fad; so that it may justly be said of, for instance, the anti-vivisectionist, that he is concerned not so much to save from pain the animals experimented on as to give pain to the experimenters. He condemns with ferocity the "vivisection" of a rat, even though the vivisection may be but the infliction of a scratch or a prick; but he glories in practising moral vivisection on his opponent. He is a sentimentalist and a humanitarian, and has all the cruelty of these characters.

What particular fad the faddist may adopt is largely a matter of accident. He is a faddist by temperament, and his mind is so constituted that to him a fad of one kind or another is a necessity; but it does not much matter what the fad is. Any opinion or mode of conduct that is unusual and can be converted into a fad is as good as any other, and in fact the faddist seldom limits himself to a single fad. Often he cultivates half a dozen. It seems a stupid thing to do, and the imperviousness of the faddist to reason seems an indication of stupidity; but the faddist is not necessarily stupid. Many are, no doubt; and of course faddism implies a certain silliness, but though he is necessarily silly, the faddist is not necessarily stupid. Some faddists are very clever in their folly, and their ingenuity in evading and distorting the arguments of their antagonists is often quite vulpine; but they never possess the ability to attain by its means the distinction for which they crave. It is this craving for distinction that is at the root of faddism. The faddist is ambitious. He craves for distinction, but has not the ability to attain sufficient distinction to satisfy him, so he is obliged to content himself with the second best; and if he cannot attain distinction he will at any cost attain singularity, and this may lead to notoriety. The adoption of a fad is an easy way of attaining, not distinction, but singularity, and the notoriety, little or much, which singularity gives. This is not distinction, but it is the nearest approach to distinction that is within reach.

The faddist is usually an idle person with an active mind. He has no occupation, or what occupation he has is insufficient to occupy his active but narrow mind; and his fad is an outlet for the part of his activity that is unoccupied. It is his hobby. He takes to it as another person takes to chess, or a third to collecting butterflies. It saves him from *ennui*. It supplies him with an interest in life, and with congenial occupation. It gives him an excuse for a pose of superiority, and to some extent satisfies his craving for distinction. It is largely because faddism is the occupation of the idle that it has pretty well disappeared since the war broke out. The faddists have found something better to do.

